# Modeling biodiversity benchmarks in variable environments

**DOI:** 10.1002/eap.1970

**Published:** 2019-07-30

**Authors:** Jian D. L. Yen, Josh Dorrough, Ian Oliver, Michael Somerville, Megan J. McNellie, Christopher J. Watson, Peter A. Vesk

**Affiliations:** ^1^ School of BioSciences The University of Melbourne Parkville VIC 3010 Australia; ^2^ ARC Centre of Excellence for Environmental Decisions The University of Melbourne Parkville VIC 3010 Australia; ^3^ Office of Environment and Heritage GPO Box 39 Sydney NSW 2001 Australia; ^4^ Fenner School of Environment and Society Frank Fenner Building, Building 141 Linnaeus Way, The Australian National University Acton ACT 2601 Australia

**Keywords:** Australia, best‐on‐offer benchmarks, biodiversity offsets, indicators, reference conditions, species richness, vegetation restoration

## Abstract

Effective environmental assessment and management requires quantifiable biodiversity targets. Biodiversity benchmarks define these targets by focusing on specific biodiversity metrics, such as species richness. However, setting fixed targets can be challenging because many biodiversity metrics are highly variable, both spatially and temporally. We present a multivariate, hierarchical Bayesian method to estimate biodiversity benchmarks based on the species richness and cover of native terrestrial vegetation growth forms. This approach uses existing data to quantify the empirical distributions of species richness and cover within growth forms, and we use the upper quantiles of these distributions to estimate contemporary, “best‐on‐offer” biodiversity benchmarks. Importantly, we allow benchmarks to differ among vegetation types, regions, and seasons, and with changes in recent rainfall. We apply our method to data collected over 30 yr at ~35,000 floristic plots in southeastern Australia. Our estimated benchmarks were broadly consistent with existing expert‐elicited benchmarks, available for a small subset of vegetation types. However, in comparison with expert‐elicited benchmarks, our data‐driven approach is transparent, repeatable, and updatable; accommodates important spatial and temporal variation; aligns modeled benchmarks directly with field data and the concept of best‐on‐offer benchmarks; and, where many benchmarks are required, is likely to be more efficient. Our approach is general and could be used broadly to estimate biodiversity targets from existing data in highly variable environments, which is especially relevant given rapid changes in global environmental conditions.

## Introduction

Biodiversity benchmarks (hereafter, benchmarks) are routinely used in a range of assessment and management applications as the quantitative estimates of desirable biodiversity states (e.g., restoration ecology [Hobbs and Harris 2001], offsetting schemes [Bull et al. 2014a, b]). Despite debate on how best to apply these benchmarks (Suding 2011, Maron et al. 2012), it is agreed that natural resource managers require transparent and repeatable methods to quantify desirable and undesirable biodiversity states (Oliver et al. 2002, 2007, Quétier and Lavorel 2011, Pardo et al. 2012, Bull et al. 2014a, b). Deviations from benchmarks provide an estimate of the quality of a site and indicate the potential for improvements in biodiversity at a site (Sinclair et al. 2002).

Benchmarks often represent quantitative estimates of “historical” (e.g., pre‐intensive agriculture, presettlement; Stoddard et al. 2006), “long undisturbed” (Parkes et al. 2003, Stoddard et al. 2006), or unmodified (Gibbons et al. 2008) reference states. However, it can be challenging to define long‐undisturbed or historical reference states because biodiversity records rarely predate human disturbances (Hobbs et al. 2010, Balaguer et al. 2014) and patterns and intensities of historical disturbances are poorly quantified. Identifying long‐undisturbed reference states is further complicated by the need to specify the time period over which baseline levels of disturbance are defined. In addition, even where long‐undisturbed reference states are known, these states may be unattainable in contemporary and future systems due to ongoing changes in species composition, climate, and landscape configuration (Pauly 1995, Millar et al. 2007, Corlett 2016). Conceptually, the use of long‐undisturbed reference states assumes that maximum biodiversity value occurs in undisturbed systems. However, maximum biodiversity value can arise at intermediate levels of disturbance (e.g., intermittent fire or livestock grazing; Shea et al. 2004).

### Best‐on‐offer biodiversity benchmarks

An alternative to a long‐undisturbed reference state is a “best‐on‐offer” reference state (Eyre et al. 2015) that represents the biodiversity values that exist under contemporary conditions (Eyre et al. 2015). Our primary focus in this paper is the development and application of benchmarks that describe best‐on‐offer reference states. Specifically, we focus on a “more‐is‐better” approach (e.g., more species or more habitat), wherein the distribution of available biodiversity states is used to set a minimum threshold for different biodiversity metrics. This approach draws on definitions of biological integrity, which often emphasize locations with many native species, few nonnative species, few overly dominant species, and high levels of functional diversity within or among species (Brooks et al. 1998, Oliver et al. 2014). Locations with high values of biodiversity metrics are expected to be associated with higher biodiversity values, increased resistance to external stressors, and reliable provision of key ecosystem services (Hobbs et al. 2010). Importantly, a focus on contemporary biodiversity states avoids the need to characterize past disturbances (natural and anthropogenic) at large spatial extents.

Best‐on‐offer reference states can be defined through expert elicitation, with experts commonly asked to identify either the values of biodiversity metrics that collectively define a best‐on‐offer state or existing sites that reflect best‐on‐offer reference states (Hiers et al. 2012, Sinclair et al. 2015). Although expert elicitation can quantify abstract and complex reference states without knowledge of past disturbances, expert assessments are inherently subjective (Sinclair et al. 2015). Similarly, the identification of reference sites relies on expert experience, knowledge, and ability to recognize when a site closely resembles the desirable reference state (Eyre et al. 2011).

An alternative to expert elicitation is to use empirical data to characterize the distribution of available states, and to use value judgements to specify desirable regions of this empirical distribution. Calculating benchmarks from data defines contemporary best‐on‐offer reference states transparently and aligns benchmarks directly with field methods, which may not always match an expert's conceptual knowledge of biodiversity. Increasing availability of archived data (Bruelheide et al. 2019) allows data‐driven benchmarks to be calculated rapidly over large spatial extents, a process that would be expensive and time‐consuming using expert elicitation.

### Estimating best‐on‐offer benchmarks over large spatial scales

Estimating benchmarks over large spatial scales is challenging because most ecological systems are highly variable in space and time (Bull et al. 2013, Kirkman et al. 2013). Attempts to define a best‐on‐offer reference state should reflect climatic and biogeographical variation at local and regional scales (Sinclair et al. 2002, Harris et al. 2006). Benchmarks that do not account for spatial or temporal variation risk under‐ or over‐valuing particular reference states depending on the time of sampling and contemporary climatic conditions. Identifying spatial and temporal variation in reference states is especially important given potential differences between past, contemporary, and future environmental conditions. The use of data‐driven benchmarks is particularly valuable in this context because benchmarks can be updated as new data are collected or made available.

Benchmarks could be estimated for many different biodiversity metrics, such as species richness, relative abundance or cover, habitat complexity, and functional diversity. Species richness is a common biodiversity metric because it is widely understood, easily measured for better known taxonomic groups, and has close associations with measures of ecological integrity, such as resistance, resilience, and ecosystem functioning (Hooper et al. 2005). Relative abundance and cover are alternatives to species richness that account for differences in dominance and rarity among species or functional groups (Yen et al. 2017a). Differences in relative abundances and cover affect the functional roles performed by different species, and can influence the demographic processes (e.g., survival, reproduction) that determine long‐term population or community persistence (Mace et al. 2008, Stuart‐Smith et al. 2013). Together, species richness and cover or abundance data provide complementary information on the nature of vegetation structure, composition, and function and often are used as practical surrogates for biodiversity more generally (Quétier and Lavorel 2011).

We sought to develop a statistical method to estimate spatially and temporally variable benchmarks from existing floristic plot data collected at a large spatial extent. We used a multivariate, hierarchical Bayesian model to estimate distributions of native species richness and summed cover (hereafter, cover) at ~35,000 sites spanning broad climatic, biogeographical, and disturbance gradients in southeastern Australia (~800,000 km^2^). We used quantiles of these distributions to define best‐on‐offer benchmarks for each of six plant growth forms (grasses, forbs, ferns, shrubs, trees, not otherwise classified [e.g., twiners, grass trees, palms]; see Oliver et al. 2019) in each combination of vegetation type, biogeographical region (bioregion), and month, for three prior rainfall amounts (below average, average, above average) based on the preceding 12 months (Dudney et al. 2017). This approach defines best‐on‐offer reference states as those locations with high native species richness and cover relative to other locations within the same vegetation type and bioregion. The multivariate, hierarchical model structure accounted for dependencies among growth forms and allowed information from extensively sampled vegetation types and bioregions to inform parameter estimates in sparsely sampled vegetation types and bioregions. We compared our modeled benchmarks to existing expert‐elicited benchmarks that were available for a small subset of the vegetation types included in our present study.

## Methods

### Study region

We used data spanning the state of New South Wales in south‐eastern Australia. The study region contains eight major climate zones, including arid, temperate, subtropical, and alpine (Stern et al. 2000). Vegetation across New South Wales is classified into 99 vegetation classes nested in 16 vegetation formations (Keith 2004). Vegetation formations are primarily distinguished by structural attributes and vegetation classes are defined by shared floristic and structural features (Keith 2004). We had data from 95 vegetation classes in 16 vegetation formations. The study region also has been classified into 18 biogeographical regions (bioregions) as part of the Interim Biogeographic Regionalization of Australia (IBRA) (Thackway and Cresswell 1995). Bioregions differ in climate, substrate, and soils but do not explicitly differ in vegetation type (Thackway and Cresswell 1995), so that a single vegetation class may occur in multiple bioregions. All vegetation survey plots used in this study had been previously allocated to a vegetation class as part of state‐wide vegetation mapping and classification (*available online*).^1^

### Vegetation data

We used full species inventories from floristic plots surveyed between 1976 and 2016 to estimate species richness and cover. From a data set of ~60,000 plots we identified 35,615 plots for species richness and 35,493 plots for cover that had been sampled using a standard 400 m^2^, typically 20 × 20 m quadrat. Some plots were surveyed multiple times, so that there were a total of 36,543 surveys for richness and 36,372 surveys for cover. Of the 1,710 theoretical combinations of bioregion (18) and vegetation class (95), we had floristic plot data for 469 combinations (27%). Many of the unobserved combinations do not exist and are unlikely to exist under current climatic conditions (e.g., subtropical rainforests in the Australian Alps bioregion).

Cover was visually estimated for each species at each plot. Cover was estimated quantitatively (0–100%) in 6,789 plots and was recorded on various Braun‐Blanquet cover‐abundance (BBCA) ordinal scales for the remaining 29,583 plots. We previously used a beta regression model to estimate quantitative cover from BBCA data, using a subset (2,809 plots) of the 6,789 true cover estimates (McNellie et al. 2019), and transformed all BBCA data to quantitative estimates accordingly. Cover estimates of trees were based on crown cover, foliage cover, or projective foliage cover (Walker and Hopkins 1990). We included the method of cover assessment, recorded for 26,130 plots, as a covariate in preliminary models. However, we did not include cover assessment method in final models because it was unknown for 10,242 plots and 95% credible intervals for the effects of cover assessment method overlapped zero.

Prior to estimating species richness and cover, we allocated all native taxa to one of six growth forms: ferns, forbs, grasses and grass‐like (hereafter, grasses), shrubs, trees, and not otherwise classified (hereafter, other; Oliver et al. 2019). Growth form was attributed based on a standardized species list for species endemic to New South Wales (Oliver et al. 2019). We calculated species richness and cover of each growth form within each plot by summing over all native species assigned to that growth form (Appendix S1: Figs S1–S2). This approach resulted in some summed cover estimates exceeding 100% due to species within growth forms having overlapping foliage. The majority of cover values were <100% (Appendix S1: Fig. S2). We calculated summed percent cover of nonnative species, irrespective of their growth form, with this same approach.

### Rainfall data

We used gridded daily rainfall totals from the Australian Water Availability Project (Jones et al. 2009) to calculate accumulated rainfall in the 12 months prior to each survey (rainfall data *available online*).^2^ These data are at a resolution of 0.05° × 0.05° (approximately 5 × 5 km). Rainfall values were interpolated from gridded data to survey location and the rainfall time series was extracted for the 12 months prior to a given survey. The 12‐month accumulated rainfall was calculated at each plot location and date. This approach was also used to calculate 3‐month and 36‐month accumulated rainfall but we did not include these variables in our models because both were positively correlated with 12‐month accumulated rainfall (*r *=* *0.70 and 0.89, respectively).

We used rainfall as a predictor variable in fitted models and used estimates of below average (10th quantile), average (50th quantile), and above average (90th quantile) rainfall to estimate benchmarks at different rainfall levels (see *Calculating species richness and cover benchmarks*, below). Given that only 469 (27%) of the theoretical combinations of bioregion and vegetation class were observed, we needed to estimate rainfall levels in the remaining 1,241 combinations. We used rainfall data from 1900 to 2015 to calculate 10th, 50th, and 90th quantiles of rainfall in the 12 months prior to the survey month at each of the initial ~60,000 plots (prior to filtering to those with complete floristics surveys). We used these three quantile estimates as response variables in separate linear regressions, with bioregion and vegetation class included as independent (additive) predictor variables. This model structure allowed us to estimate 10th, 50th, and 90th rainfall quantiles in unobserved combinations of bioregion and vegetation class. Links to model data and code are in *Supporting information*.

### Data analysis: overview

Our aim was to develop a statistical method to estimate benchmarks from existing floristic plot data. Specifically, we related data on species richness and cover of six growth forms to vegetation class, bioregion, month, rainfall, and nonnative cover to estimate distributions of species richness and cover in each month in each combination of bioregion and vegetation class at three rainfall levels. We used quantiles from this distribution to define benchmarks, that is, best‐on‐offer biodiversity states.

We used a multivariate, hierarchical Bayesian model to model all growth forms simultaneously, but fitted separate models for native species richness and cover. Our model accounts for correlations in species richness and cover among growth forms, and allows for differences in species richness and cover among vegetation classes, bioregions, and months. We assumed that vegetation class and bioregion had additive effects on species richness and cover, and nested the effects of vegetation classes within those of the broader vegetation formations. The model also included effects of rainfall and percentage cover of nonnative species, as well as stochastic variation in species richness among years and plots.

Vegetation classes are partially based on systematic differences in the species richness and cover of different growth forms. We expected these systematic differences among vegetation classes to capture many of the correlations among growth forms. For example, shifts from tree cover in forests to grass cover in grasslands are reflected in different vegetation classes. Our multivariate model also accounted for residual correlations among growth forms. The model structure reflects our expectation that growth forms are not independent within vegetation classes. For example, species richness and cover might be positively correlated among growth forms due to shared responses to local environmental conditions (e.g., soil nutrient availability, recent rainfall). By contrast, species richness and cover might be negatively correlated among growth forms due to competitive interactions within a single vegetation type (e.g., trees and grasses; Scholes and Archer 1997).

Our model included cover of nonnative vegetation as a predictor variable but did not explicitly include prior anthropogenic disturbance, for which reliable data were unavailable. Nonnative vegetation cover is often positively correlated with the frequency and intensity of disturbances related to landscape transformations such as agriculture and urbanisation (Hobbs 2001) and invasion by nonnative species can modify vegetation composition, structure, and function (Simberloff et al. 2013).

We allowed rainfall effects to differ among bioregions. Although we expected generally positive effects of rainfall, the magnitude of this effect on richness and cover might vary systematically owing to broad differences in climate and landform. For example, we expected that small increases in total rainfall would have larger effects in arid and semiarid regions than in coastal regions that generally experience higher rainfall amounts.

We modeled monthly trends in two ways. First, we estimated smooth monthly trends and allowed these trends to differ among bioregions. This model form incorporates seasonal variation in species richness and cover and reflects our expectation that seasonal patterns differ throughout the study region due to broad differences among arid, temperate, alpine, and subtropical climatic zones. Second, we included months as random intercepts in our model but did not allow monthly variation to differ among bioregions. This model form is less ecologically realistic but has 186 fewer parameters to estimate than the dynamic model, which was expected to improve model predictions in bioregions with few plots.

### Data analysis: model definition

We defined the vector **y**_*i*_ as the species richness and the vector **z**_*i*_ as the cover of all growth forms in survey *i* (six‐dimensional vectors). We assumed that species richness was Poisson distributed and that cover was lognormally distributed. We accounted for dependencies among growth forms by including multivariate normal over‐dispersion on the log‐transformed location parameters for species richness and cover. The model was

$$y_{i}∼\text{Poisson}\left(\lambda_{i}\right)\left[\text{for species richness}\right]$$

or

$$\log\left(z_{i}+0.001\right)∼\text{Normal}\left(\log\left(\lambda_{i}\right),\sigma \right)\left[\text{for cover}\right];$$

$$\log\left(\lambda_{i}\right)∼\;\text{MVN}\left(\mu_{i},\Sigma \right);$$

$$\mu_{i}=\alpha +\beta_{v(i)}+\gamma_{m(i),b(i)}+r_{i}\delta_{b(i)}+e_{i}\epsilon +\zeta_{y(i)}+\eta_{p(i)};$$

where **Σ** is the covariance matrix for all growth forms, *v*(*i*) is the vegetation class of plot *i*,* m*(*i*) is the month of plot *i*,* b*(*i*) is the IBRA bioregion of plot *i*,* r*_*i*_ is the amount of rainfall in the 12 months preceding a survey in plot *i*,* e*_*i*_ is the proportional nonnative cover recorded in plot *i*,* y*(*i*) is the year in which plot *i* was surveyed, and *p*(*i*) is the identity of plot *i*. Parameters in boldface type are six‐dimensional vectors with one value for each growth form, so that **α** is a six‐dimensional vector containing average log(richness) or log(cover) for each growth form, **β** is a vector of deviations from the intercept in vegetation class *v*(*i*), **γ** is a vector of deviations in bioregion *b*(*i*) in month *m*(*i*), **δ** is a vector of regression coefficients for the effect of rainfall in bioregion *b*(*i*), **ϵ** is a vector of regression coefficients for the effect of nonnative cover, **ζ** is a vector of deviations in year *y*(*i*), and **η** is a vector of deviations in plot *p*(*i*). The tilde (~) denotes “distributed as.” MVN is the multivariate normal distribution. The log(*z *+* *0.001) transformation for cover data is equivalent to a Box‐Cox transformation with parameters λ_1_ = 0 and λ_2_ = 0.001 (Box and Cox 1964).

We used the above model form in models with regionally varying monthly trends. We used a simplified linear predictor in models with a regionally consistent monthly effect. This model included separate terms for bioregion and month, and the linear predictor was

$$\mu_{i}=\alpha +\beta_{v(i)}+\gamma_{b(i)}+r_{i}\delta_{b(i)}+e_{i}\epsilon +\zeta_{y(i)}+\eta_{p(i)}+\kappa_{m(i)};$$

where **κ** is a vector of deviations from average log(richness) or log(cover) in month *m*(*i*), **β** is a vector of deviations in bioregion *b*(*i*), and all other terms are as defined above.

### Data analysis: prior distributions

In models with regionally varying monthly trends, we used a random walk prior for months within bioregions, **γ**_*m*,*b*_, to account for the non‐independence of species richness and cover between months. We used a first‐order random walk for all months from August to May, so that **γ** values were normally distributed with mean equal to the preceding month:

$$\gamma_{m,b}∼\text{Normal}\left(\gamma_{\left[m-1\right],b},\sigma_{b}\right).$$

We began the random walk in July, so that **γ** values in July were normally distributed with zero mean

$$\gamma_{\mathrm{July},b}∼\text{Normal}\left(0,\sigma_{b}\right)$$

and we linked June to May and July to ensure that **γ** values changed smoothly between these months

$$\gamma_{\mathrm{June},b}∼\;\text{Normal}\left(\left[\gamma_{\mathrm{May},b}+\gamma_{\mathrm{July},b}\right]/2,\sigma_{b}\right).$$

In models with regionally consistent monthly effects, we used hierarchical priors for the effects of bioregion and month, with the normally distributed deviations in each bioregion and month:

$$\gamma_{b}∼\text{Normal}\left(0,\sigma_{b}\right);\kappa_{m}∼\text{Normal}\left(0,\sigma_{m}\right).$$

In all models, we used a hierarchical prior for the effects of vegetation class, with deviations from mean log(richness) or log(cover) in each vegetation class assumed to be normally distributed with a mean common to the vegetation formation to which that vegetation class belongs:

$$\beta_{v(i)}∼\text{Normal}\left(\theta_{f(v(i))},\sigma_{v}\right).$$

The common mean for the vegetation class was assigned a zero‐mean, exchangeable normal prior distribution:

$$\theta_{f(v(i))}∼\text{Normal}\left(0,\sigma_{f}\right).$$

We assigned a hierarchical prior to the bioregion‐specific effects of rainfall, and the common mean for rainfall effects was assigned a vague, zero‐mean normal prior distribution:

$$\delta_{b(i)}∼\text{Normal}\left(\iota ,\sigma_{r}\right);\iota ∼\text{Normal}\left(0,10\right).$$

We assigned a vague, zero‐mean normal prior distribution to the effects of nonnative species cover:

$$\epsilon ∼\text{Normal}\left(0,10\right).$$

We assigned exchangeable, zero‐mean normal prior distributions to the effects of year and plot:

$$\zeta_{y(i)}∼\text{Normal}\left(0,\sigma_{y}\right);\eta_{p(i)}∼\text{Normal}\left(0,\sigma_{p}\right).$$

All standard deviations were six‐dimensional vectors and each element was assigned a half‐normal prior distribution:

$$\sigma_{v},\sigma_{f},\sigma_{b},\sigma_{r},\sigma_{y},\sigma_{p},\sigma_{m}∼\text{HalfNormal}\left(0,2\right).$$

We used an LKJ prior for the covariance matrix **Σ** (Lewandowski et al. 2009). The LKJ prior is governed by a single parameter *a*, whose value determines the prior weight assigned to different correlation matrices. Setting *a* equal to 1 assigns equal weight to all correlation matrices, while increasing this parameter above 1 places more weight on the unit diagonal correlation matrix. Specifically, the density of the LKJ prior is proportional to the determinant of the correlation matrix raised to the power of the parameter *a* (i.e. *p*(**Φ**) = |**Φ**|^*a*^, where **Φ** is the correlation matrix). The full covariance matrix **Σ** can be recovered by multiplying **Φ** by a vector of standard deviations, **σ**_*s*_. We set *a* equal to 4 and assigned **σ**_*s*_ a half‐normal prior distribution with mean 0 and standard deviation equal to 2.

### Computational details

We fitted the above model in Stan version 2.12 (Carpenter et al. 2017, Stan Development Team 2017a) using the rstan R package (version 2.12.1; Stan Development Team 2017b) in R version 3.4.0 (R Core Team 2017). Stan is general purpose software for Bayesian models, and uses Hamiltonian Monte Carlo to generate samples from the posterior distribution more efficiently than many other MCMC methods (e.g., Metropolis‐Hastings; Hoffman and Gelman 2014). All inferences were based on four chains of 5,000 iterations following a 5,000 iteration burn‐in period. We assessed model convergence using Gelman‐Rubin statistics (Gelman and Rubin 1992) and the effective number of samples, *n*_eff_. All Rhats were <1.01 and *n*_eff_ was greater than 1,000 for all parameters. Links to model code and data are in *Supporting information*.

### Calculating species richness and cover benchmarks

We used quantiles of marginal posterior distributions to estimate benchmarks. We note that any quantile could be used as a benchmark value; here, we estimated the 55th, 65th, and 75th quantiles to represent lower through to higher benchmark values.

Our multivariate model accounted for correlated residuals among growth forms in model fitting but our use of marginal posterior distributions assumed that the posterior quantiles of species richness or cover are independent among growth forms, conditional on vegetation class, bioregion, month, rainfall, and nonnative species cover. We did not use full posterior quantiles because multivariate quantiles have multiple dimensions (six dimensions in our case) and cannot be summarized as a single number for each growth form (Chaudhuri 1996).

We calculated species richness and cover benchmarks in two ways. First, we used models with regionally varying monthly trends to calculate dynamic benchmarks: one value for each growth form in each combination of vegetation class, bioregion, and month, at each of three rainfall levels (below average, average, above average). This approach resulted in 20,520 dynamic species richness and cover benchmarks for each growth form and rainfall level (738,720 in total). Second, we used models with regionally consistent monthly trends to calculate static benchmarks: one value for each growth form in each combination of vegetation class and bioregion using the average rainfall for each combination of bioregion and vegetation class. This approach resulted in 1,710 static species richness and cover benchmarks for each growth form (20,520 in total). We assumed a best‐on‐offer reference state would exclude the presence of nonnative plant species and so all estimated benchmarks were based on 0% nonnative plant species cover. In our data set, minimum nonnative cover was 0% in 69% of observed combinations of bioregion and vegetation class, and minimum nonnative cover was <5% in 83% of observed combinations.

### Comparison of modeled best‐on‐offer benchmarks with expert‐elicited benchmarks

We compared estimated static benchmarks (available for 1,710 combinations of bioregion and vegetation class) to benchmarks derived for four combinations of bioregion and vegetation class through a separate expert elicitation study (Dorrough et al. 2019 and *Supporting information*). We note that expert‐elicited benchmarks were not obtained for the purpose of validating our modeling approach but, rather, were used opportunistically to assess whether modeled benchmarks were broadly consistent with expert knowledge of these vegetation classes. There were several differences in definitions between elicited and modeled benchmarks. In particular, expert‐elicited benchmarks were derived for spring (October) in a median rainfall year and cover estimates were defined as visual estimates of growth‐form cover. Visual estimates of growth‐form cover differ from the plot data used to derive modeled benchmarks, where cover was estimated from summed species‐cover estimates. Also, rather than estimating the 75th percentile, experts were asked to estimate the range of values they would expect among sites that were in a best‐on‐offer reference state in the contemporary landscape. Expert‐elicited benchmarks were available for Inland Floodplain Woodlands in the Riverina bioregion, Sand Plain Mulga Shrublands in the Mulga Lands bioregion, Subtropical Rainforests in the South Eastern Queensland bioregion, and Western Slopes Grassy Woodlands in the Brigalow Belt South bioregion.

## Results

### Patterns in modeled species richness and cover

Estimated mean native species richness was highest in forbs, shrubs, and grasses and lowest in trees, ferns, and growth forms not otherwise classified (others; Table 1). Rainfall was positively associated with species richness of all growth forms (5–24% increase in species richness for each standard deviation increase in rainfall) with the strongest associations with ferns, forbs, and others and the weakest with trees. The cover of nonnative species had a weak, negative association with species richness of all growth forms (1–12% decrease in species richness for each standard deviation increase in nonnative cover; Table 1).

**Table 1:** Estimated mean values of native species richness and cover and multiplicative effects of rainfall amount and percentage cover of nonnative species

	Ferns	Forbs	Grasses	Other	Shrubs	Trees
Species richness						
Mean	1	6	5	1	6	2
Rainfall	1.19	1.20	1.16	1.24	1.16	1.05
Nonnative species cover	0.95	0.99	0.98	0.96	0.88	0.96
Cover						
Mean	0.05	2.63	8.25	0.19	6.43	3.01
Rainfall	1.90	1.45	1.66	2.17	1.22	1.11
Nonnative species cover	1.1	1.1	1.19	1.18	0.97	1.07

*Notes*

Mean values and rainfall effects are averages over all bioregions and vegetation classes. Effects of rainfall and percentage nonnative cover are multiplicative. For example, the value of 1.19 in the first column of the second row reflects a 19% increase in species richness for each one standard deviation increase in rainfall, whereas the value of 0.95 in the first column of the third row reflects a 5% decrease in species richness for each one standard deviation increase in nonnative species cover. Values shown are from models with regionally consistent monthly trends. Links to estimates for all model parameters are in *Supporting information*.

**Figure 1:**
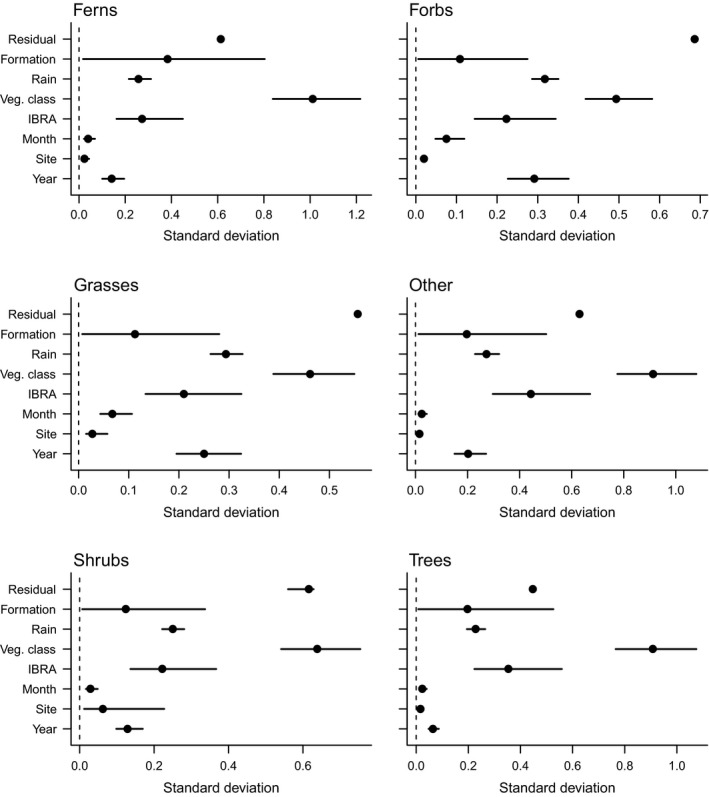
Standard deviations of model coefficients. Values are proportional to the amount of variation in native species richness explained by a given variable. Residual is the variation not explained by the variables included in a given model. Veg., vegetation; IBRA, Interim Biogeographic Regionalisation of Australia. Error bars show 95% credible intervals.

Fitted models explained 20–55% of the variation in observed species richness (links to model fit statistics are in *Supporting information*). There was substantial variation in species richness among vegetation classes with less variation among bioregions and vegetation formations (Fig. 1). Estimated mean native species richness of forbs and grasses differed more among years than among months (Fig. 1). Estimated associations were very similar in static and dynamic models of species richness. Links to full parameter estimates are in *Supporting information*.

Estimated mean cover was highest in shrubs and grasses and lowest in ferns and others (Table 1). Rainfall was strongly positively associated with cover of all growth forms (11–117% increase in cover for each standard deviation increase in rainfall) with strongest associations with ferns, forbs, grasses, and others (Table 1). The cover of nonnative species also had a positive association with the cover of all growth forms with the exception of shrubs (7–19% increase in cover for each standard deviation increase in nonnative cover; Table 1).

**Figure 2:**
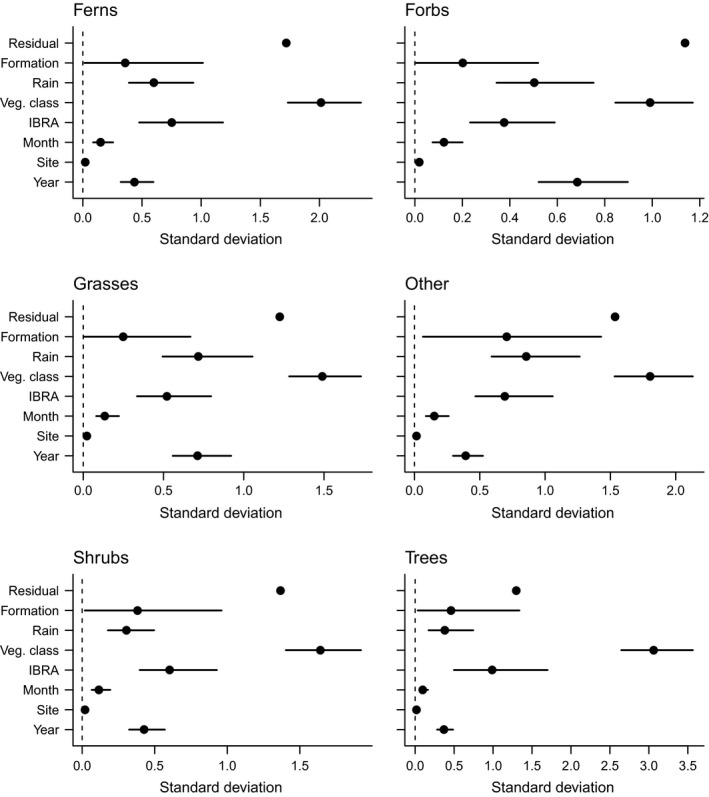
Standard deviations of model coefficients. Values are proportional to the amount of variation in native species cover explained by a given variable. Residual is the variation not explained by the variables included in a given model.

Fitted models explained 6–20% of the variation in observed summed cover (links to model fit statistics are in *Supporting information*). There was substantial variation in cover among vegetation classes with less variation among bioregions and vegetation formations (Fig. 2). Estimated mean native cover differed among years, with forbs and grasses differing more among years than other growth forms (Fig. 2). Estimated associations were similar in static and dynamic models of cover.

### Correlations among growth forms

Residual species richness was positively correlated among all growth forms in static and dynamic models. In dynamic models, correlation coefficients (*r*) ranged from 0.12 to 0.95 (mean = 0.53; Table 2). The highest correlations were between forbs and grasses (0.95) and between others and ferns (0.94; Table 2). The lowest correlations were between shrubs and forbs (0.16) and between trees and grasses (0.12; Table 2). Tree species richness was strongly correlated with that of ferns (0.76), shrubs (0.76), and others (0.89; Table 2). Correlations among growth forms in static models did not differ from those in the dynamic model by more than 0.02.

**Table 2:** Correlations among growth forms in richness (lower triangular) and cover (upper triangular)

Richness	Cover					
	Ferns	Forbs	Grasses	Other	Shrubs	Trees
Ferns		**0.18**	**0.08**	**0.17**	**0.09**	**0.08**
Forbs	**0.62**		**0.36**	**0.21**	**0.07**	**0.03**
Grasses	**0.44**	**0.95**		**0.08**	**–0.04**	–0.01
Other	**0.94**	**0.55**	**0.41**		**0.16**	**0.14**
Shrubs	**0.35**	**0.16**	**0.21**	**0.58**		**0.15**
Trees	**0.76**	**0.21**	**0.12**	**0.89**	**0.76**	

*Notes*

Estimated correlations are based on dynamic models. Boldface type denotes values with 95% credible intervals not overlapping zero.

Residual cover was mostly positively correlated among growth forms in static and dynamic models. In dynamic models, correlation coefficients (*r*) ranged from –0.04 to 0.36 (mean = 0.12) (Table 2). Grasses were the exception, with negative correlations with trees (*r = –*0.01) and shrubs (*r = –*0.04). All other pairs of growth forms were positively correlated, with the highest correlations between grasses and forbs (*r *=* *0.36) and others and forbs (*r *=* *0.21; Table 2). Correlations among growth forms in static models did not differ from those in the dynamic model by more than 0.01.

### Comparison of modeled benchmarks with expert‐elicited benchmarks

**Figure 3:**
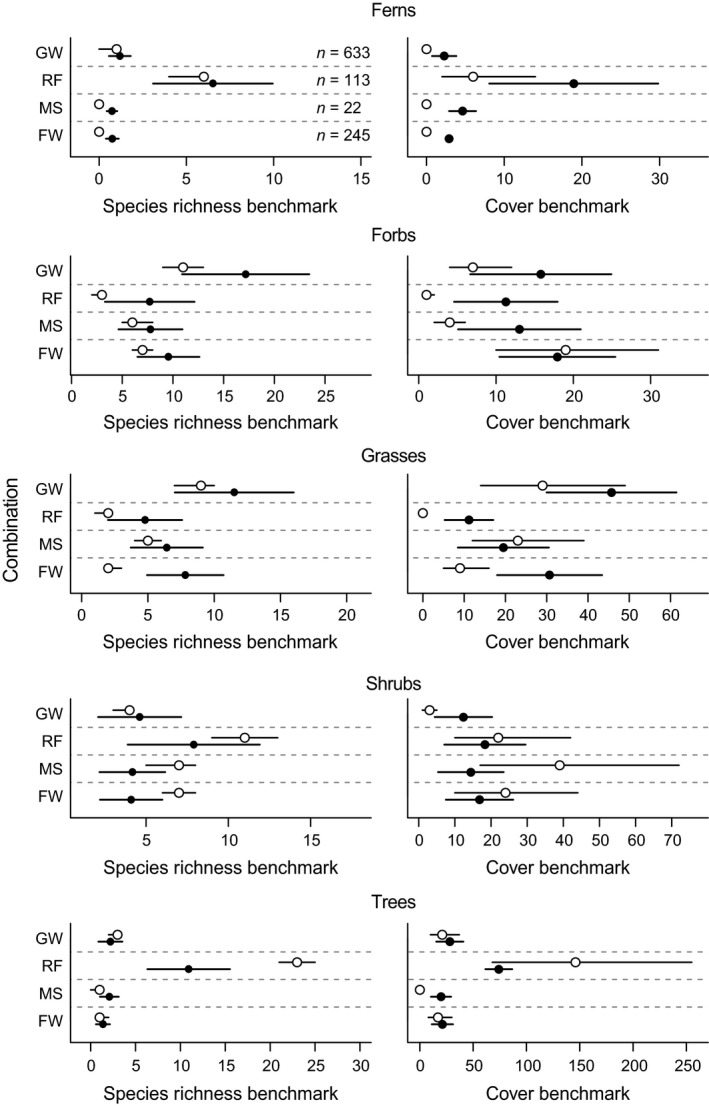
Comparison of expert‐elicited benchmarks (solid points) and modeled benchmarks (open points) for four combinations of bioregion and vegetation class. Solid points are mean elicited values and bars on these points extend from 10th to 90th percentiles of best‐on‐offer biodiversity states. Open points are 65th percentiles and bars on these points extend from 55th to 75th percentiles from fitted models. Numbers in the far‐left segment are the number of plots surveyed in each combination (FW, Riverina, Inland Floodplain Woodlands; MS, Mulga Lands, Sand Plain Mulga Shrublands; RF, South Eastern Queensland, Subtropical Rainforests; GW, Brigalow Belt South, Western Slopes Grassy Woodlands).

Modeled benchmarks for both richness and cover aligned closely with expert‐elicited benchmarks for all growth forms (Fig. 3). Differences between modeled and elicited benchmarks were most pronounced in forbs and grasses, where modeled benchmarks tended to be lower than elicited benchmarks, and in rainforest tree and shrub cover in Mulga Shrublands, where modeled benchmarks were higher than elicited benchmarks (Fig. 3). The number of plots underlying modeled benchmarks was not consistently associated with the difference between modeled and elicited benchmarks (Fig. 3).

### Patterns in modeled benchmarks

Species richness benchmarks were highest in shrubs and forbs, followed by trees, others, grasses, and ferns (Appendix S2: Figs S1–S3). Cover benchmarks were highest in trees, shrubs, and other growth forms, followed by ferns, grasses, and forbs (Appendix S2: Figs S4–S6). We note that summed cover could exceed 100% in our data set due to overlapping foliage among species within a growth form. Modeled cover benchmarks were highest and most variable in combinations of bioregion, vegetation class, and month for which we had few observations, and this variability increased from lower to higher benchmark quantiles (Appendix S2: Figs S4–S6).

**Figure 4:**
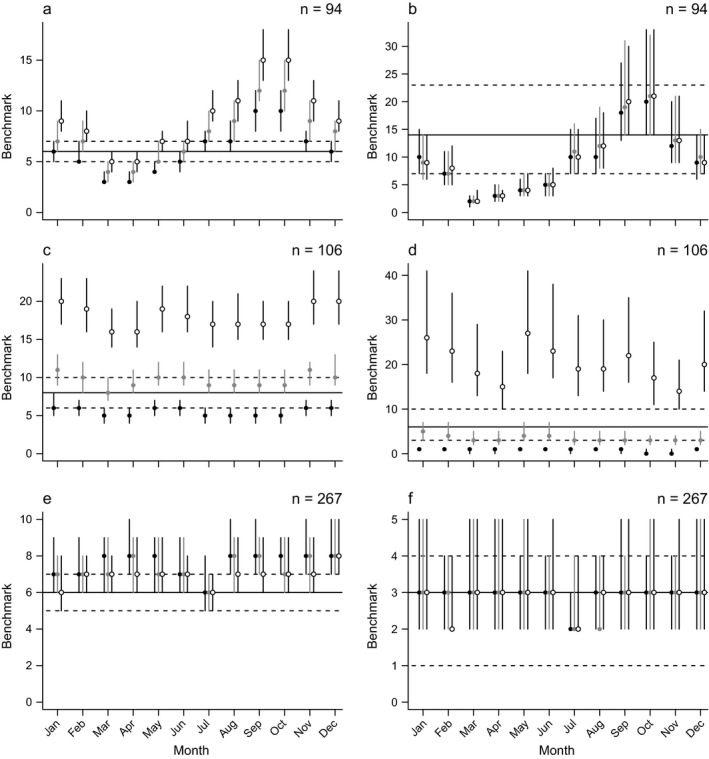
Estimated benchmarks for forb (a, c, e) species richness and (b, d, f) cover. Horizontal lines are static benchmarks based on 65th percentiles (solid lines) bounded by 55th and 75th percentiles (dashed lines). Points are dynamic benchmarks at three different rainfall levels: below average (solid black points), average (solid gray points), and above average (open points). Points are 65th percentiles and vertical bars extend from 55th to 75th percentiles. Sample sizes in the top‐right corner are the number of plots surveyed in a given combination of bioregion and vegetation class (a, b, Riverina, Floodplain Transition Woodlands; c, d, Nandewar, Northern Tableland Dry Sclerophyll Forests; e, f, Sydney Basin, Coastal Floodplain Wetlands).

Static and dynamic benchmarks were broadly similar in many cases. However, richness and cover were associated with season and past rainfall in some vegetation classes, bioregions, and growth forms. The effects of season and past rainfall differed among vegetation classes and bioregions, and were most pronounced in forbs (Fig. 4). For example, forb richness and cover peaked in the austral spring in southern and central bioregions (e.g., Riverina bioregion, Fig 4a, b) but in the austral summer in northern bioregions (e.g., Brigalow Belt South, see *Supporting information*). In drier bioregions, richness and cover often had no clear seasonal trend but were associated strongly with total rainfall over the 12 months prior to a given survey (e.g., Nandewar bioregion, Fig. 4c, d). Vegetation in coastal bioregions often had weak associations with season and rainfall (e.g., coastal vegetation classes in the Sydney Basin bioregion, Fig. 4e, f). Links to plots of benchmarks for all combinations of bioregion and vegetation class with >50 plots are in *Supporting information*.

## Discussion

### A need for variable, disaggregated benchmarks

We present a statistical method to estimate best‐on‐offer (benchmark) reference states using a large number of vegetation survey plots distributed along broad climatic, biogeographical, and disturbance gradients. We demonstrated estimation of native species richness and cover benchmarks that accounted for spatial variation among vegetation types and bioregions and temporal variation among months and due to recent rainfall. Although vegetation type explained much of the variation in species richness and cover of shrubs and trees, temporally varying factors such as year and rainfall often explained substantial amounts of the variation in richness and cover of forbs, grasses, and growth forms not otherwise classified (others). Temporal variation in richness and cover often differed among vegetation classes and bioregions (e.g., spring vs. summer peaks at different latitudes), and accounting for these interactions in benchmark estimates is a key feature of our modeling approach. Dynamic benchmarks that accounted for monthly variation and recent rainfall amounts followed ecologically plausible trends in combinations of bioregion and vegetation class. Substantial variation in benchmarks among vegetation types, bioregions, years, and rainfall levels reinforces the need for benchmarks that differ both spatially and temporally (Bull et al. 2013, Kirkman et al. 2013).

We estimated benchmarks for each of six growth forms: ferns, forbs, grasses, shrubs, trees, and others. Growth forms are an intermediate grouping that simplify the complexity of full species inventories without reducing these inventories to simple biodiversity metrics such as total species richness and total abundance or cover (Mayfield et al. 2010, Maseyk et al. 2016, Yen et al. 2017b). We observed substantial variation in species richness and cover benchmarks among growth forms, which supports the use of disaggregated benchmarks in our study system. Calculating separate (disaggregated) benchmarks for each growth form tracks compositional, structural, and functional components of biodiversity, and reduces the risk that one component can substitute or “eclipse” another, for example the loss of forb species being substituted for by the addition of shrub species (McCarthy et al. 2004, Oliver et al. 2014). Accounting for variation among different components of biodiversity is critical to distinguishing sites that differ in composition, structure, or function but that otherwise have similar total numbers of species or relative cover (e.g., grasslands and rainforests).

### Expert‐elicited vs. data‐driven benchmarks

Although our estimated static benchmarks (averaged over months and rainfall amounts) were broadly consistent with expert‐elicited benchmarks for four combinations of bioregion and vegetation class, there were several differences between modeled and expert‐elicited benchmarks. Specifically, elicited benchmarks exceeded modeled benchmarks for forbs, grasses, and ferns in several vegetation classes, and modeled benchmarks exceeded elicited benchmarks for shrubs and trees in some vegetation classes (Fig. 3). Within a vegetation type, elicited benchmarks tended to exceed modeled benchmarks in low diversity growth forms (e.g., grasses in rainforests), while the opposite was true of highly diverse growth forms (e.g., trees in rainforests). These differences might be due to differences in data or differences in perceptions or interpretations of sampling scales. For example, modeled benchmarks were based on summed estimates of cover from species inventories, whereas experts were asked to consider visual estimates of growth‐form cover, which do not necessarily include species with overlapping cover. Similarly, elicited benchmarks are subject to high levels of individual bias, with a tendency to avoid very low or high estimates possibly resulting in species richness and cover being overestimated in uncommon growth forms and underestimated in diverse or common growth forms. Individual bias has been shown to affect elicited estimates of bounded values (Montibeller and von Winterfeldt 2015), including elicited abundances of rare species (Lichtenstein et al. 1978, Farmer et al. 2012).

Although it is difficult to validate biodiversity benchmarks without independent, a priori knowledge of reference states, differences between elicited and modeled benchmarks indicate that these two approaches might be complementary rather than competing. Although a requirement for many benchmarks (>700,000 values in this study) precludes the elicitation of all benchmarks, targeted elicitation might inform benchmarks in locations with few or no data or when modeled benchmarks have low precision. In these cases, elicited benchmarks might be used to define benchmarks directly or to inform prior distributions to be updated when data become available. An important consequence of modeling benchmarks from empirical data is that these benchmarks can be based on the same field methods, measurement scales, and data types commonly used in contemporary environmental assessment and management applications. Aligning benchmarks with data collection is important because empirical data will not necessarily reflect an expert's conceptualization of a given vegetation type.

### Ecological inferences from estimated benchmarks

Our method characterized variation in the richness and cover of plant growth forms among vegetation types, bioregions, seasons, and at multiple rainfall levels. Past rainfall and resulting changes in soil moisture are particularly important for fast‐growing and shallow‐rooted plants (e.g., grasses and forbs), and can substantially influence species richness and cover in these groups (Zavaleta et al. 2003). We observed strong seasonal patterns in some combinations of vegetation class and bioregion, but the nature of these patterns often differed among vegetation classes or bioregions. In many cases, benchmarks differed more among recent rainfall levels than among months, which indicates that recent rainfall explains more variation in cover and richness than season. Failing to account for variation due to recent rainfall and season could result in benchmarks that are either unattainable or too‐readily attainable, which may lead to undesirable biodiversity outcomes (Kirkman et al. 2013). Close associations with rainfall suggest that benchmarks might be sensitive to changes in natural disturbance regimes, such as drought. An advantage of variable, data‐driven benchmarks is their explicit incorporation of changing environmental conditions, with the capacity to update benchmarks as new data become available.

Our use of a multivariate model structure gives some insight into interactions among growth forms within vegetation classes and bioregions. We observed positive residual correlations between almost all pairs of growth forms, with the only exceptions being weak negative correlations between the cover of grasses and the cover of shrubs and trees (Table 2). Positive correlations might reflect local aspects of site fertility, disturbance, and weather, to which many species within growth forms respond similarly. Such an association also was observed between the cover of native and nonnative species, with highly productive locations supporting more cover of both native and nonnative species (Martín‐Forés et al. 2017). Our weak negative correlations between the cover of grasses and trees and shrubs match expectations from theoretical and empirical work on coexistence between grasses and woody vegetation (Walker et al. 1986, Scholes and Archer 1997).

### Implications of variable, data‐driven benchmarks

We estimated benchmarks as the 55th, 65th, and 75th quantiles of the marginal posterior species richness or cover distribution. A remaining challenge for practical application of our method is the identification of the most appropriate quantile for use as the best‐on‐offer reference state. Our use of quantiles explicitly assumes that many native species and high cover are more desirable than few native species and low cover relative to other locations within the same vegetation type and bioregion. We assume that more native species and higher cover are associated with higher biological integrity (Oliver et al. 2014). Under this assumption, the choice of the appropriate quantile is inherently subjective but may be guided by four considerations. First, if there is a sampling bias towards relatively undisturbed (or disturbed) vegetation, a lower (or higher) quantile may be appropriate. Second, we found that small sample sizes and large amounts of residual variation widened posterior credible intervals and potentially inflated benchmarks, and this variability increased from lower to higher quantiles, which might suggest that a lower quantile is appropriate when few data are available or when residual variation is high. For example, residual variation was higher for cover than richness in our case study, indicating that a lower quantile might be appropriate for cover benchmarks. Third, independent estimates of richness and cover benchmarks might result in high quantiles being unattainable for richness and cover simultaneously, in which case a lower benchmark might be needed to ensure that current and future sites can feasibly achieve best‐on‐offer status. Last, biodiversity benchmarks used in biodiversity offset schemes can have significant implications for offset ratios. When used to assess a potential development site, low quantiles will generally result in higher offset requirements whereas high quantiles will result in lower offset requirements. Each of these four points requires careful consideration when selecting appropriate quantiles for use in a particular benchmark application.

A related challenge for practical applications is the choice between dynamic and static benchmarks. Although dynamic benchmarks capture important temporal variation in biodiversity states, estimating dynamic benchmarks reduces the amount of data underpinning a given benchmark, with consequences for the precision of benchmark estimates and the selection of benchmark quantiles. Knowledge of ecological dynamics can inform the choice of dynamic or static benchmarks. For example, one would expect substantial variation in biodiversity states in regions that experience large fluctuations in water availability or high levels of seasonal variation. Such associations were apparent in estimated benchmarks, where dynamic benchmarks diverged from static benchmarks in spring and autumn in a highly seasonal system (Riverina Floodplain Woodlands; Fig. 4a, b) and in dry and wet years in a system with highly variable rainfall (Nandewar Dry Sclerophyll Forests; Fig. 4c, d), but not in a more stable system (Sydney Basin Coastal Woodlands; Fig. 4e, f). These differences highlight situations where dynamic benchmarks might be preferred over static benchmarks, following which the choice and implementation of dynamic or static benchmarks can be guided by data availability and knowledge of a specific benchmark application.

### Benefits of variable, data‐driven benchmarks

Our Bayesian modeling approach is easily updated, allowing benchmarks to be refined as new data become available. For example, we observed interannual variation in species richness and cover of grasses and forbs, which suggests that benchmarks for these growth forms might need updating to capture long‐term climatic trends (e.g., warming and drying climates; Parmesan and Yohe 2003). Existing benchmarks can be used to define prior distributions and newly collected data can be used to update benchmark estimates (Ellison 2004). This approach would substantially reduce the subsequent computational demands of our modeling approach because the large data set used initially (~35,000 observations for each of six growth forms) can be reduced to a relatively small set of prior distributions (~5,000 parameters). An important consideration when collecting new data to update existing benchmarks is the additional information gain from new data versus the cost of collecting such data (Canessa et al. 2015).

A focus on data‐driven benchmarks that reflect contemporary biodiversity states supports transparent and updateable benchmarks, which we believe will have broad application to conservation planning and management. Whereas benchmarks based on concepts of minimal disturbance emphasize divergences from idealized biodiversity states, our approach highlights contemporary states and existing data. The use of transparent benchmarks, linked to empirical data, is particularly beneficial in policy settings, where contentious decisions are best supported by defensible benchmarks that reflect empirical observations. The models outlined here were used to deliver benchmarks to the Biodiversity Assessment Method, which operates under the New South Wales Biodiversity Conservation Act 2016 (Office of Environment and Heritage 2018). Field testing of these benchmarks in real world applications, such as assessing biodiversity values to support biodiversity management, will provide valuable feedback on the validity of the estimated benchmarks and on key details of implementation, such as the identification of appropriate quantiles to define best‐on‐offer reference states. We believe our approach is general and could be used broadly to calculate transparent and updateable biodiversity targets in dynamic environments, especially given unprecedented and global access to ‘big data’ such as floristic inventories stored in data warehouses (Franklin et al. 2017, Bruelheide et al. 2019).

## Data Availability

Details of additional models, all data, and all model code are available from the Open Science Framework at https://doi.org/10.17605/osf.io/6p2b8

## Notes

1
http://www.environment.nsw.gov.au/research/Visclassification.htm

2
http://www.bom.gov.au/jsp/awap/

## References

[eap1970-bib-0002] Balaguer, L. , A. Escudero , J. F. Martín‐Duge , I. Mola , and J. Aronson . 2014 The historical reference in restoration ecology: re‐defining a cornerstone concept. Biological Conservation 176:12–20.

[eap1970-bib-0004] Box, G. E. P. , and D. R. Cox . 1964 An analysis of transformations. Journal of the Royal Statistical Society B 26:211–252.

[eap1970-bib-0005] Brooks, R. P. , T. J. O'Connell , D. H. Wardrop , and L. E. Jackson . 1998 Towards a regional index of biological integrity: the example of forested riparian ecosystems. Environmental Monitoring and Assessment 51:131–143.

[eap1970-bib-0006] Bruelheide, H. , et al. 2019 sPlot—a new tool for global vegetation analyses. Journal of Vegetation Science 30:161–186.

[eap1970-bib-0007] Bull, J. W. , K. B. Suttle , N. J. Singh , and E. J. Milner‐Gulland . 2013 Conservation when nothing stands still: moving targets and biodiversity offsets. Frontiers in Ecology and the Environment 11:203–210.

[eap1970-bib-0008] Bull, J. W. , A. Gordon , E. A. Law , K. B. Suttle , and E. J. Millner‐Gulland . 2014a Importance of baseline specification in evaluating conservation interventions and achieving no net loss of biodiversity. Conservation Biology 28:799–809. 24945031 10.1111/cobi.12243 PMC4241037

[eap1970-bib-0009] Bull, J. W. , E. J. Milner‐Gulland , K. B. Suttle , and N. J. Singh . 2014b Comparing biodiversity offset calculation methods with a case study in Uzbekistan. Biological Conservation 178:2–10.

[eap1970-bib-0010] Canessa, S. , G. Guillera‐Arroita , J. J. Lahoz‐Monfort , D. M. Southwell , D. P. Armstrong , I. Chadès , R. C. Lacy , and S. J. Converse . 2015 When do we need more data? A primer on calculating the value of information for applied ecologists. Methods in Ecology and Evolution 6:1219–1228.

[eap1970-bib-0011] Carpenter, B. , A. Gelman , M. D. Hoffman , D. Lee , B. Goodrich , M. Betancourt , M. Brubaker , J. Guo , P. Li , and A. Riddell . 2017 Stan: a probabilistic programming language. Journal of Statistical Software 76:1–32. 10.18637/jss.v076.i01 PMC9788645 36568334

[eap1970-bib-0012] Chaudhuri, P. 1996 On a geometric notion of quantiles for multivariate data. Journal of the American Statistical Association 91:862–872.

[eap1970-bib-0013] Corlett, R. T. 2016 Restoration, reintroduction, and rewilding in a changing world. Trends in Ecology and Evolution 31:453–462. 26987771 10.1016/j.tree.2016.02.017

[eap1970-bib-0014] Dorrough, J. , S. J. Sinclair , and I. Oliver . 2019 Expert predictions of changes in vegetation condition reveal perceived risks in biodiversity offsetting. PLoS ONE 14:e0216703. 31067268 10.1371/journal.pone.0216703 PMC6505952

[eap1970-bib-0015] Dudney, J. , L. M. Hallett , L. Larios , E. C. Farrer , E. N. Spotswood , C. Stein , and K. N. Suding . 2017 Lagging behind: have we overlooked previous‐year rainfall effects in annual grasslands? Journal of Ecology 105:484–495.

[eap1970-bib-0016] Ellison, A. M. 2004 Bayesian inference in ecology. Ecology Letters 7:509–520.

[eap1970-bib-0017] Eyre, T. J. , A. L. Kelly , and V. J. Neldner . 2011 Method for the establishment and survey of reference sites for biocondition. Queensland Herbarium, Department of Science, Information Technology and Innovation, Brisbane (Queensland), Australia. https://www.qld.gov.au/environment/assets/documents/plants-animals/biodiversity/reference-sites-biocondition.pdf

[eap1970-bib-0018] Eyre, T. J. , A. L. Kelly , V. J. Neldner , B. A. Wilson , D. J. Ferguson , M. J. Laidlaw , and A. J. Franks . 2015 BioCondition: a condition assessment framework for terrestrial biodiversity in Queensland, assessment manual version 2.2. Queensland Herbarium, Department of Science, Information Technology, Innovation and Arts Brisbane, Queensland, Australia.

[eap1970-bib-0019] Farmer, R. G. , M. L. Leonard , and A. G. Horn . 2012 Observer effects and avian‐call‐count survey quality: rare‐species biases and overconfidence. Auk 129:76–86.

[eap1970-bib-0020] Franklin, J. , J. M. Serra‐Diaz , A. D. Syphard , and H. M. Regan . 2017 Big data for forecasting the impacts of global change on plant communities. Global Ecology and Biogeography 26:6–17.

[eap1970-bib-0021] Gelman, A. , and D. B. Rubin . 1992 Inference from iterative simulation using multiple sequences. Statistical Science 7:457–472.

[eap1970-bib-0022] Gibbons, P. , S. V. Briggs , D. A. Ayers , S. Doyle , J. Seddon , C. McElhinny , N. Jones , R. Sims , and J. S. Doody . 2008 Rapidly quantifying reference conditions in modified landscapes. Biological Conservation 141:2483–2493.

[eap1970-bib-0023] Harris, J. A. , R. J. Hobbs , E. Higgs , and J. Aronson . 2006 Ecological restoration and global climate change. Restoration Ecology 14:170–176.

[eap1970-bib-0024] Hiers, J. K. , R. J. Mitchell , A. Barnett , J. R. Walters , M. Mack , B. Williams , and R. Sutter . 2012 The dynamic reference concept: measuring restoration success in a rapidly changing no‐analogue future. Ecological Restoration 30:27–36.

[eap1970-bib-0025] Hobbs, R. J. 2001 Synergisms among habitat fragmentation, livestock grazing, and biotic invasions in southwestern Australia. Conservation Biology 15:1522–1528.

[eap1970-bib-0026] Hobbs, R. J. , and J. A. Harris . 2001 Restoration ecology: repairing the Earth's ecosystems in the new millennium. Restoration Ecology 9:239–246.

[eap1970-bib-0027] Hobbs, R. J. , et al. 2010 Guiding concepts for park and wilderness stewardship in an era of global environmental change. Frontiers in Ecology and the Environment 8:483–490.

[eap1970-bib-0028] Hoffman, M. D. , and A. Gelman . 2014 The No‐U‐Turn Sampler: adaptively setting path lengths in Hamiltonian Monte Carlo. Journal of Machine Learning Research 15:1351–1381.

[eap1970-bib-0029] Hooper, D. U. , et al. 2005 Effects of biodiversity on ecosystem functioning: a consensus of current knowledge. Ecological Monographs 75:3–35.

[eap1970-bib-0030] Jones, D. , W. Wang , and R. Fawcett . 2009 High‐quality spatial climate datasets for Australia. Australian Meteorological Magazine 58:233–248.

[eap1970-bib-0031] Keith, D. A. 2004 Ocean shores to desert dunes: the native vegetation of NSW and the ACT. Department of the Environment and Conservation, Sydney, New South Wales, Australia.

[eap1970-bib-0032] Kirkman, L. K. , A. Barnett , B. W. Williams , J. K. Hiers , S. M. Pokswinski , and R. J. Mitchell . 2013 A dynamic reference model: a framework for assessing biodiversity restoration goals in a fire‐dependent ecosystem. Ecological Applications 23:1574–1587. 24261041 10.1890/13-0021.1

[eap1970-bib-0034] Lewandowski, D. , D. Kurowicka , and H. Joe . 2009 Generating random correlation matrices based on vines and extended onion method. Journal of Multivariate Analysis 100:1989–2001.

[eap1970-bib-0035] Lichtenstein, S. , P. Slovic , B. Fischhoff , M. Layman , and B. Combs . 1978 Judged frequency of lethal events. Journal of Experimental Psychology: Human Learning and Memory 4:551–578. 731196

[eap1970-bib-0036] Mace, G. M. , N. J. Collar , K. J. Gaston , C. Hilton‐Taylor , H. R. Akçakaya , N. Leader‐Williams , E. J. Millner‐Gulland , and S. N. Stuart . 2008 Quantification of extinction risk: IUCN's system for classifying threatened species. Conservation Biology 22:1424–1442. 18847444 10.1111/j.1523-1739.2008.01044.x

[eap1970-bib-0037] Maron, M. , R. J. Hobbs , A. Moilanen , J. W. Matthews , K. Christie , T. A. Gardner , D. A. Keith , D. B. Lindenmayer , and C. A. McAlpine . 2012 Faustian bargains? Restoration realities in the context of biodiversity offset policies. Biological Conservation 155:141–148.

[eap1970-bib-0038] Martín‐Forés, I. , G. R. Guerin , and A. J. Lowe . 2017 Weed abundance is positively correlated with native plant diversity in grasslands of southern Australian. PLoS ONE 12:e0178681. 28570604 10.1371/journal.pone.0178681 PMC5453567

[eap1970-bib-0039] Maseyk, F. J. F. , L. P. Barea , R. T. T. Stephens , H. P. Possingham , G. Dutson , and M. Maron . 2016 A disaggregated biodiversity offset accounting model to improve estimation of ecological equivalency and no net loss. Biological Conservation 204:322–332.

[eap1970-bib-0040] Mayfield, M. M. , S. P. Bonser , J. W. Morgan , I. Aubin , S. McNamara , and P. A. Vesk . 2010 What does species richness tell us about functional trait diversity? Predictions and evidence for responses of species and functional trait diversity to land‐use change. Global Ecology and Biogeography 19:423–431.

[eap1970-bib-0041] McCarthy, M. A. , et al. 2004 The Habitat Hectares approach to vegetation assessment: an evaluation and suggestions for improvement. Ecological Management and Restoration 5:24–27.

[eap1970-bib-0042] McNellie, M. J. , J. Dorrough , and I. Oliver . 2019 Species abundance distributions should underpin ordinal cover‐abundance transformations. Applied Vegetation Science 22:361–372

[eap1970-bib-0043] Millar, C. , N. L. Stephenson , and S. L. Stephens . 2007 Climate change and forests of the future: managing in the face of uncertainty. Ecological Applications 17:2145–2151. 18213958 10.1890/06-1715.1

[eap1970-bib-0044] Montibeller, G. , and D. von Winterfeldt . 2015 Cognitive and motivational biases in decision and risk analysis. Risk Analysis 35:1230–1251. 25873355 10.1111/risa.12360

[eap1970-bib-0045] Office of Environment and Heritage . 2018 The biodiversity assessment method. http://www.environment.nsw.gov.au/biodiversity/assessmentmethod.htm

[eap1970-bib-0046] Oliver, I. , P. L. Smith , I. Lunt , and D. Parkes . 2002 Pre‐1750 vegetation, naturalness and vegetation condition: what are the implications for biodiversity conservation? Ecological Management and Restoration 3:176–178.

[eap1970-bib-0047] Oliver, I. , H. Jones , and D. L. Schmoldt . 2007 Expert panel assessment of attributes for natural variability benchmarks for biodiversity. Austral Ecology 32:453–475.

[eap1970-bib-0048] Oliver, I. , D. J. Eldridge , C. Nadolny , and W. K. Martin . 2014 What do site condition multi‐metrics tell us about species biodiversity? Ecological Indicators 38:262–271.

[eap1970-bib-0049] Oliver, I. , M. J. McNellie , G. Steenbeeke , L. Copeland , M. F. Porteners , and J. Wall . 2019 Expert allocation of primary growth form to the NSW flora underpins the Biodiversity Assessment Method. Australasian Journal of Environmental Management 26:124–136.

[eap1970-bib-0050] Pardo, I. , et al. 2012 The European reference condition concept: a scientific and technical approach to identify minimally‐impacted river ecosystems. Science of the Total Environment 420:33–42. 22326313 10.1016/j.scitotenv.2012.01.026

[eap1970-bib-0051] Parkes, D. , G. Newell , and D. Cheal . 2003 Assessing the quality of native vegetation: the habitat hectares approach. Ecological Management and Restoration 4:S29–S38.

[eap1970-bib-0052] Parmesan, C. , and G. Yohe . 2003 A globally coherent fingerprint of climate change impacts across natural systems. Nature 421:37–42. 12511946 10.1038/nature01286

[eap1970-bib-0053] Pauly, D. 1995 Anecdotes and the shifting baseline syndrome of fisheries. Trends in Ecology and Evolution 10:430–430. 21237093 10.1016/s0169-5347(00)89171-5

[eap1970-bib-0054] Quétier, F. , and S. Lavorel . 2011 Assessing ecological equivalence in biodiversity offset schemes: key issues and solutions. Biological Conservation 144:2991–2999.

[eap1970-bib-0055] R Core Team . 2017 R: a language and environment for statistical computing. R Foundation for Statistical Computing, Vienna, Austria https://www.R-project.org

[eap1970-bib-0056] Scholes, R. J. , and S. R. Archer . 1997 Tree‐grass interactions in savannas. Annual Review of Ecology and Systematics 28:517–544.

[eap1970-bib-0057] Shea, K. , S. H. Roxburgh , and E. S. Rauschert . 2004 Moving from pattern to process: coexistence mechanisms under intermediate disturbance regimes. Ecology Letters 7:491–508.

[eap1970-bib-0058] Simberloff, D. , et al. 2013 Impacts of biological invasions: what's what and the way forward. Trends in Ecology and Evolution 28:58–66. 22889499 10.1016/j.tree.2012.07.013

[eap1970-bib-0059] Sinclair, A. R. E. , S. A. R. Mduma , and P. Arcese . 2002 Protected areas as biodiversity benchmarks for human impact: agriculture and the Serengeti avifauna. Proceedings of the Royal Society B 269:2401–2405. 12495481 10.1098/rspb.2002.2116 PMC1691175

[eap1970-bib-0060] Sinclair, S. J. , P. Griffioen , D. H. Duncan , J. E. Millett‐Riley , and M. D. White . 2015 Quantifying ecosystem quality by modeling multi‐attribute expert opinion. Ecological Applications 25:1463–1477. 26552257 10.1890/14-1485.1

[eap1970-bib-0061] Stan Development Team . 2017a Stan modeling language users guide and reference manual. Version 2.12.0. http://mc-stan.org

[eap1970-bib-0062] Stan Development Team . 2017b RStan: the R interface to Stan. R package version 2.12.1. http://mc-stan.org

[eap1970-bib-0063] Stern, H. , G. Hoedt , and J. Ernst . 2000 Objective classification of Australian climates. Australian Meteorological Magazine 49:87–96.

[eap1970-bib-0064] Stoddard, J. L. , D. P. Larsen , C. P. Hawkins , R. K. Johnson , and R. H. Norris . 2006 Setting expectations for the ecological condition of streams: the concept of reference condition. Ecological Applications 16:1267–1276. 16937796 10.1890/1051-0761(2006)016[1267:seftec]2.0.co;2

[eap1970-bib-0065] Stuart‐Smith, R. D. , et al. 2013 Integrating abundance and functional traits reveals new global hotspots of fish diversity. Nature 501:539–542. 24067714 10.1038/nature12529

[eap1970-bib-0066] Suding, K. N. 2011 Toward an era of restoration in ecology: successes, failures, and opportunities ahead. Annual Review of Ecology, Evolution, and Systematics 42:465–487.

[eap1970-bib-0067] Thackway, R. , and I. D. Cresswell . 1995 An interim biogeographic regionalisation for Australia: a framework for setting priorities in the National Reserves System Cooperative Program, Version 4.0. Australian Nature Conservation Agency, Canberra, Australian Capital Territory. Australia.

[eap1970-bib-0068] Walker, J. , and M. S. Hopkins . 1990 Vegetation Pages 58–86 *in* McDonald R. C., Isbell R. F., Speight J. G., and Walker J., editors. Australian soil and land survey field handbook. CSIRO, Canberra, Australian Capital Territory, Australia.

[eap1970-bib-0069] Walker, J. , J. A. Robertson , L. K. Penridge , and P. J. H. Sharpe . 1986 Herbage response to tree thinning in a *Eucalyptus crebra* woodland. Austral Ecology 11:135–140.

[eap1970-bib-0070] Yen, J. D. L. , J. R. Thomson , J. M. Keith , D. M. Paganin , and R. Mac Nally . 2017a How do different aspects of biodiversity change through time? A case study on an Australian bird community. Ecography 40:642–650.

[eap1970-bib-0071] Yen, J. D. L. , J. R. Thomson , J. M. Keith , D. M. Paganin , E. Fleishman , D. S. Dobkin , J. M. Bennett , and R. Mac Nally . 2017b Balancing generality and specificity in ecological gradient analysis with species abundance distributions and individual size distributions. Global Ecology and Biogeography 26:318–322.

[eap1970-bib-0072] Zavaleta, E. S. , M. R. Shaw , N. R. Chiariello , B. D. Thomas , E. E. Cleland , C. B. Field , and H. A. Mooney . 2003 Grassland responses to three years of elevated temperature, CO2, precipitation, and N deposition. Ecological Monographs 73:585–604.

