# Supplementary material for: Modeling biodiversity benchmarks in variable environments
Source: Ecol Appl. 2019 Jul 30;29(7):e01970. doi: 10.1002/eap.1970 (PMC6852130; doi:10.1002/eap.1970)
Supplement: Supplementary file 1 [file EAP-29-na-s001.pdf]

Supporting Information. Jian D. L. Yen, Josh Dorrough, Ian Oliver, Michael Somerville, Megan J. McNellie, Christopher J. Watson, Peter A. Vesk. 2019. Modelling biodiversity benchmarks in variable environments. *Ecological Applications*.

Appendix S1: Supporting figures

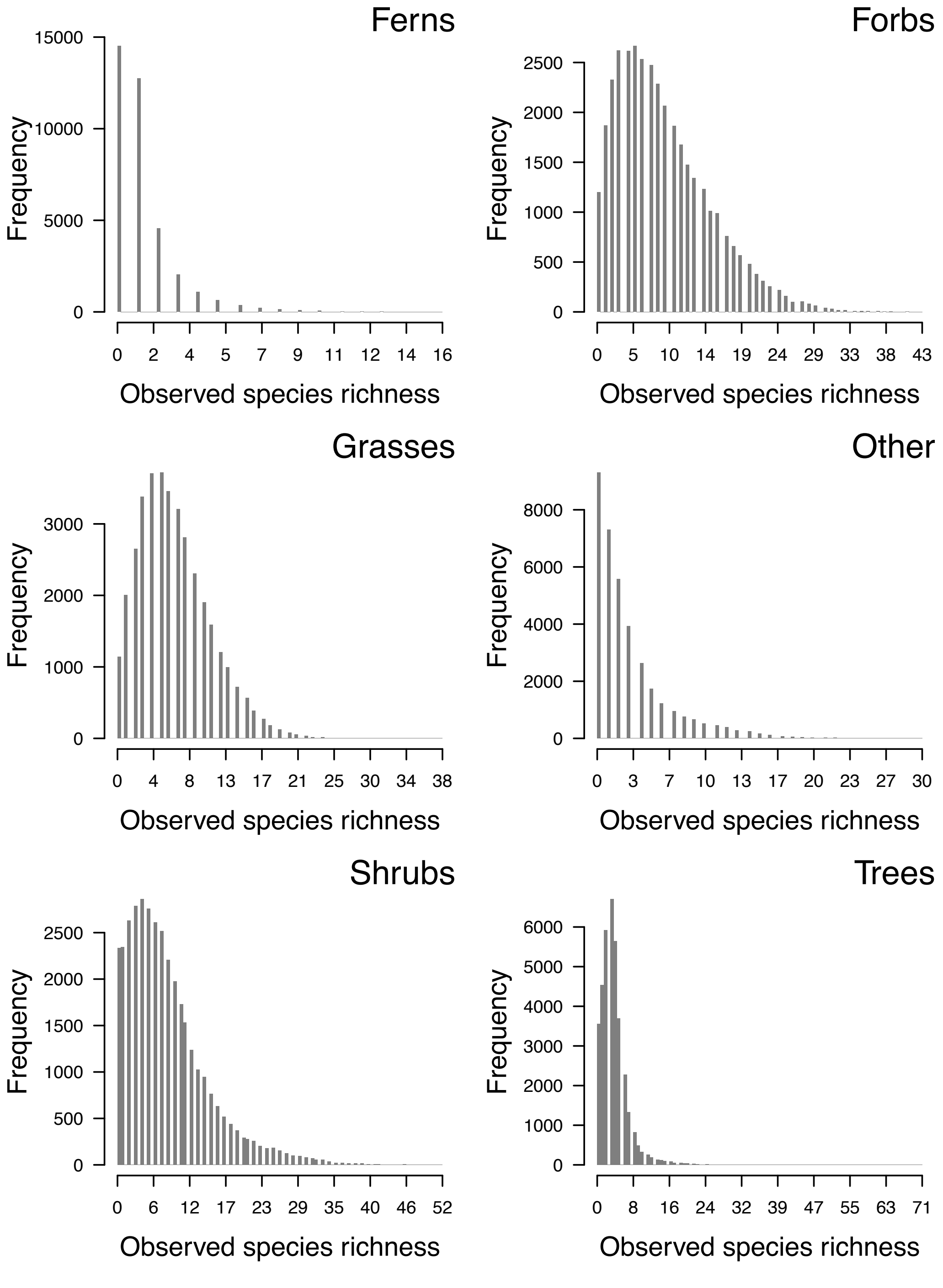

Fig. S1. Distribution of observed native species richness in each growth form.

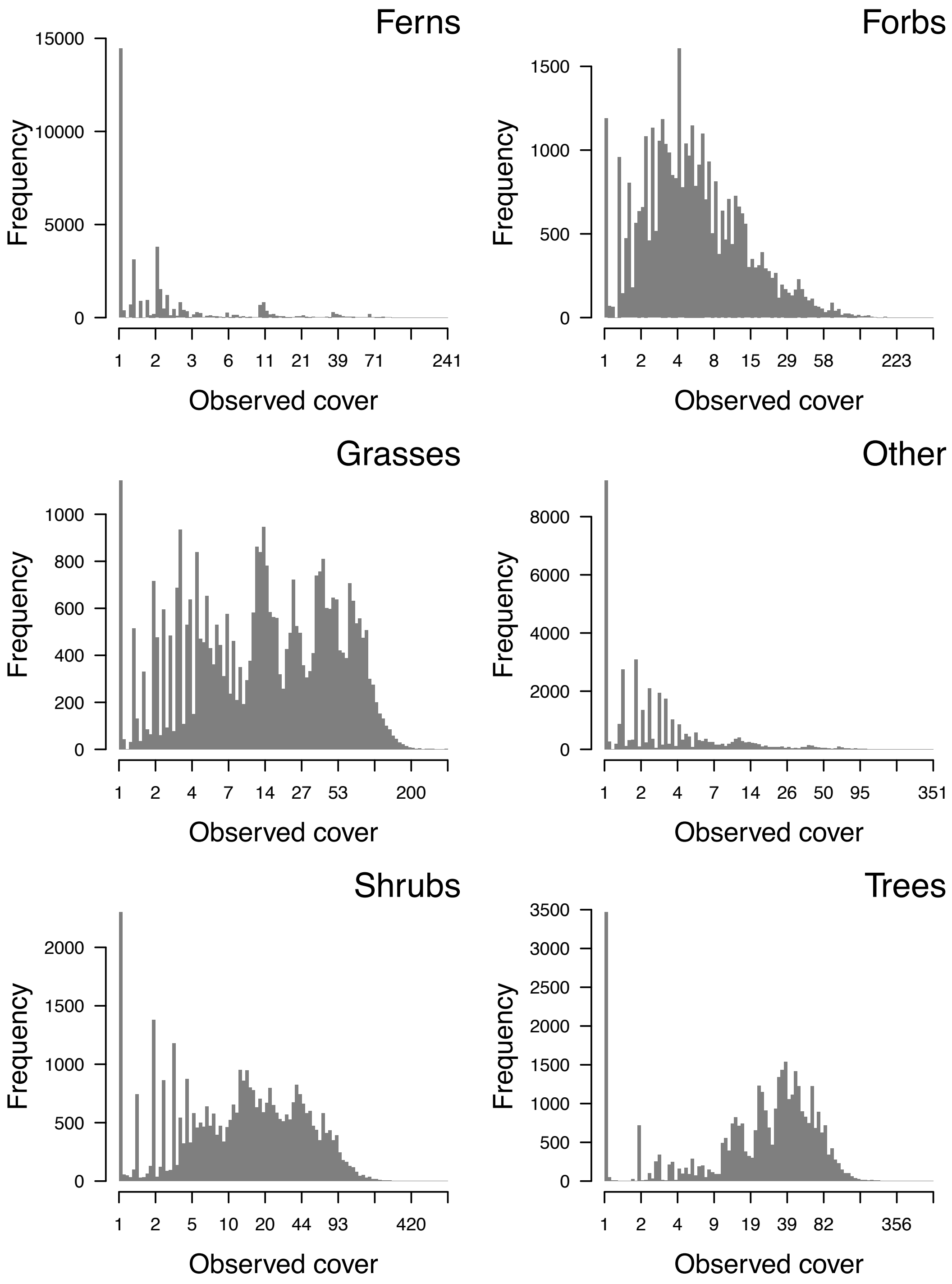

Fig. S2. Distribution of observed native species cover in each growth form.
