# Supplementary material for: Modeling biodiversity benchmarks in variable environments
Source: Ecol Appl. 2019 Jul 30;29(7):e01970. doi: 10.1002/eap.1970 (PMC6852130; doi:10.1002/eap.1970)
Supplement: Supplementary file 2 [file EAP-29-na-s002.pdf]

Supporting Information. Jian D. L. Yen, Josh Dorrough, Ian Oliver, Michael Somerville, Megan J. McNellie, Christopher J. Watson, Peter A. Vesk. 2019. Modelling biodiversity benchmarks in variable environments. *Ecological Applications*.

Appendix S2: Supporting figures

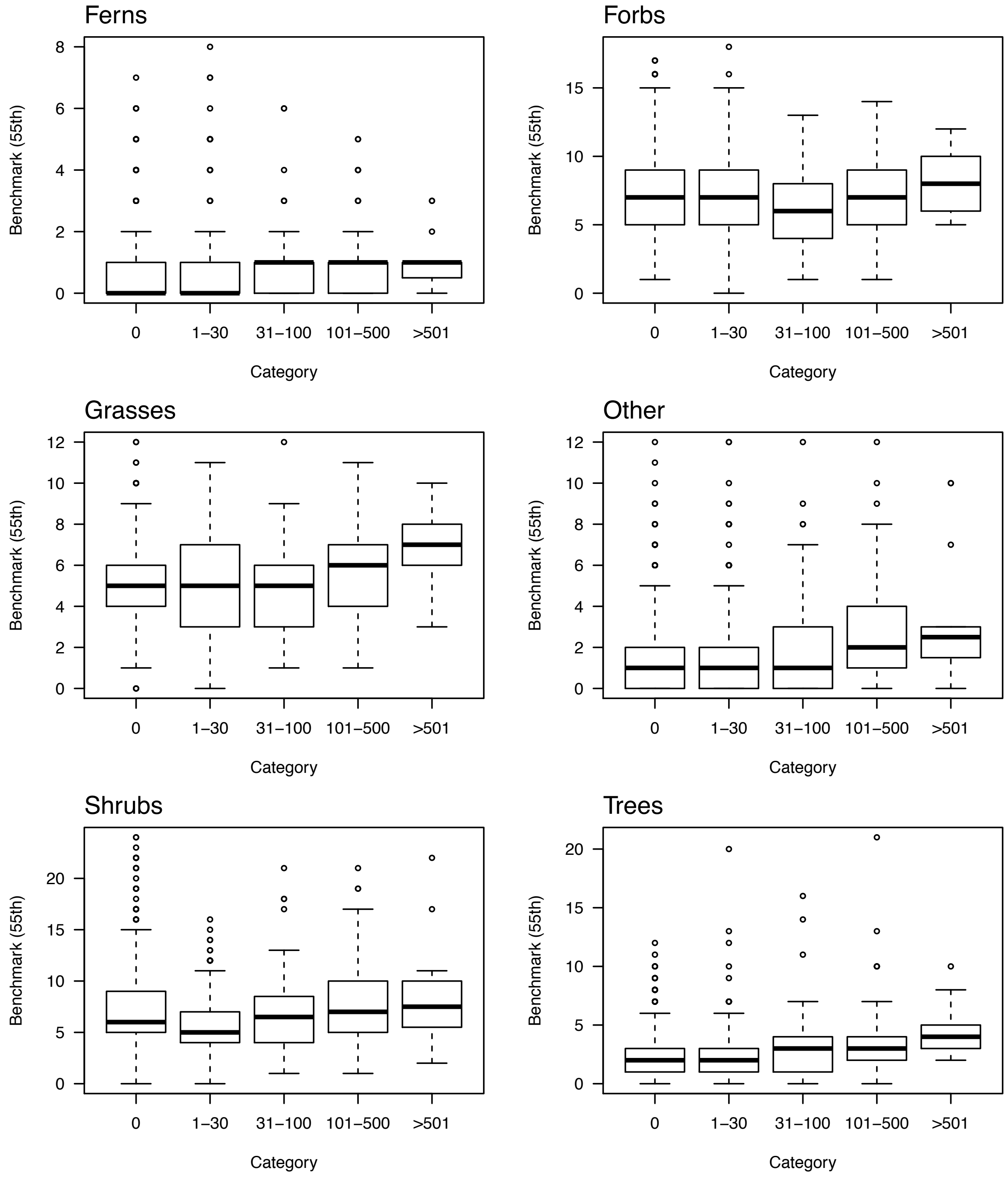

Fig. S1. Estimated native species richness benchmarks (55th percentiles) plotted against number of plots in each combination of bioregion and vegetation class.

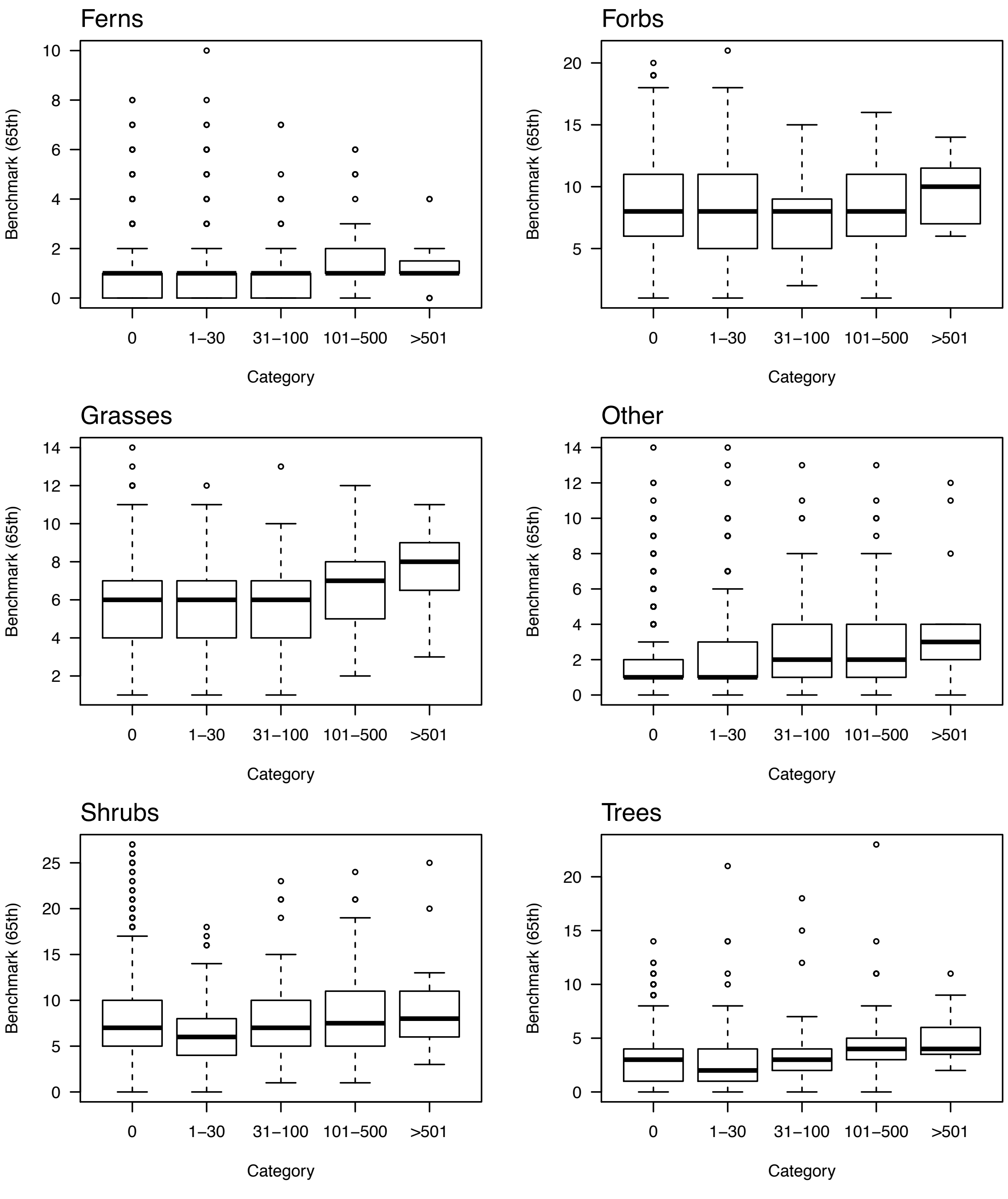

Fig. S2. Estimated native species richness benchmarks (65th percentiles) plotted against number of plots in each combination of bioregion and vegetation class.

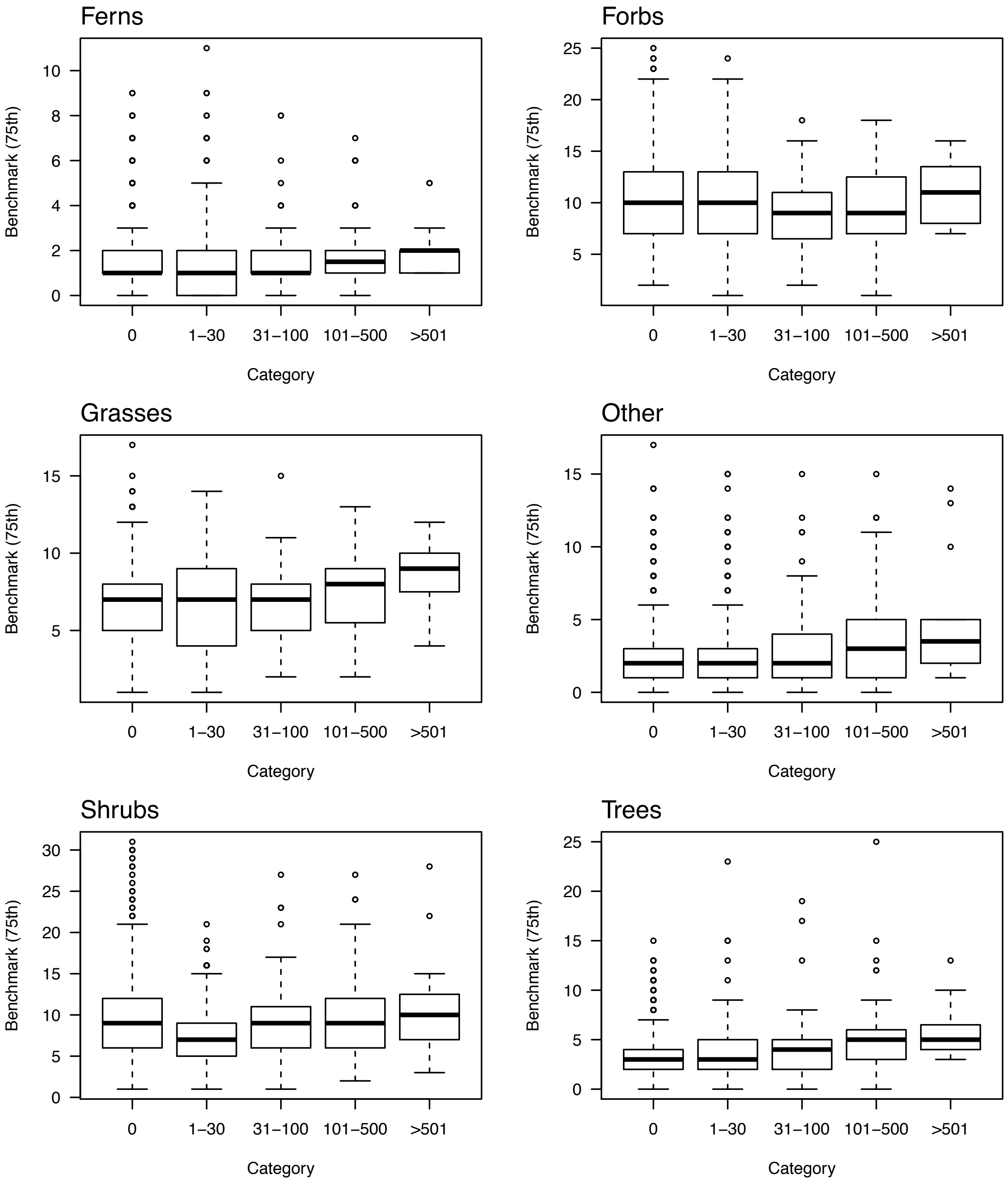

Fig. S3. Estimated native species richness benchmarks (75th percentiles) plotted against number of plots in each combination of bioregion and vegetation class.

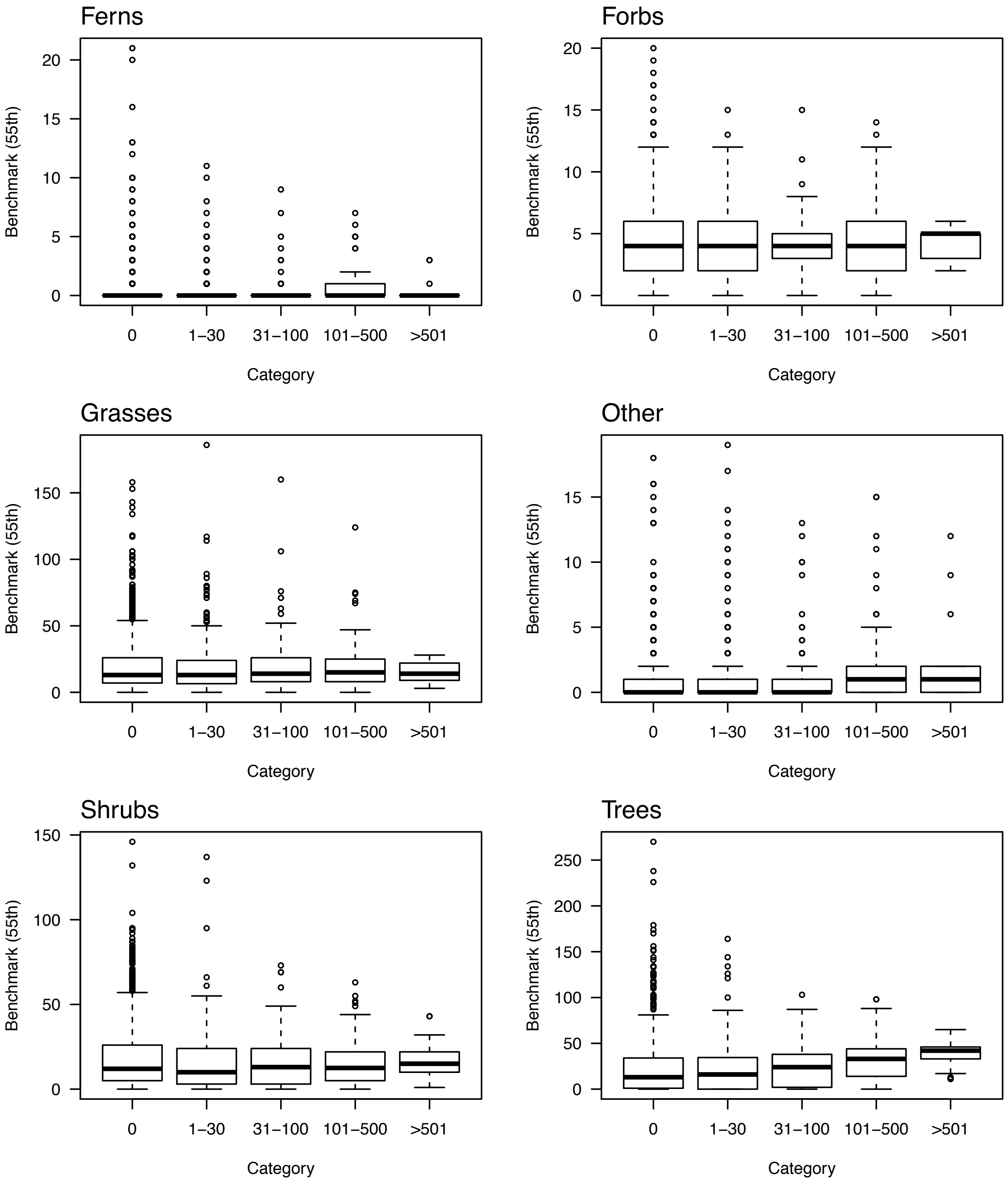

Fig. S4. Estimated native species cover benchmarks (55th percentiles) plotted against number of plots in each combination of bioregion and vegetation class.

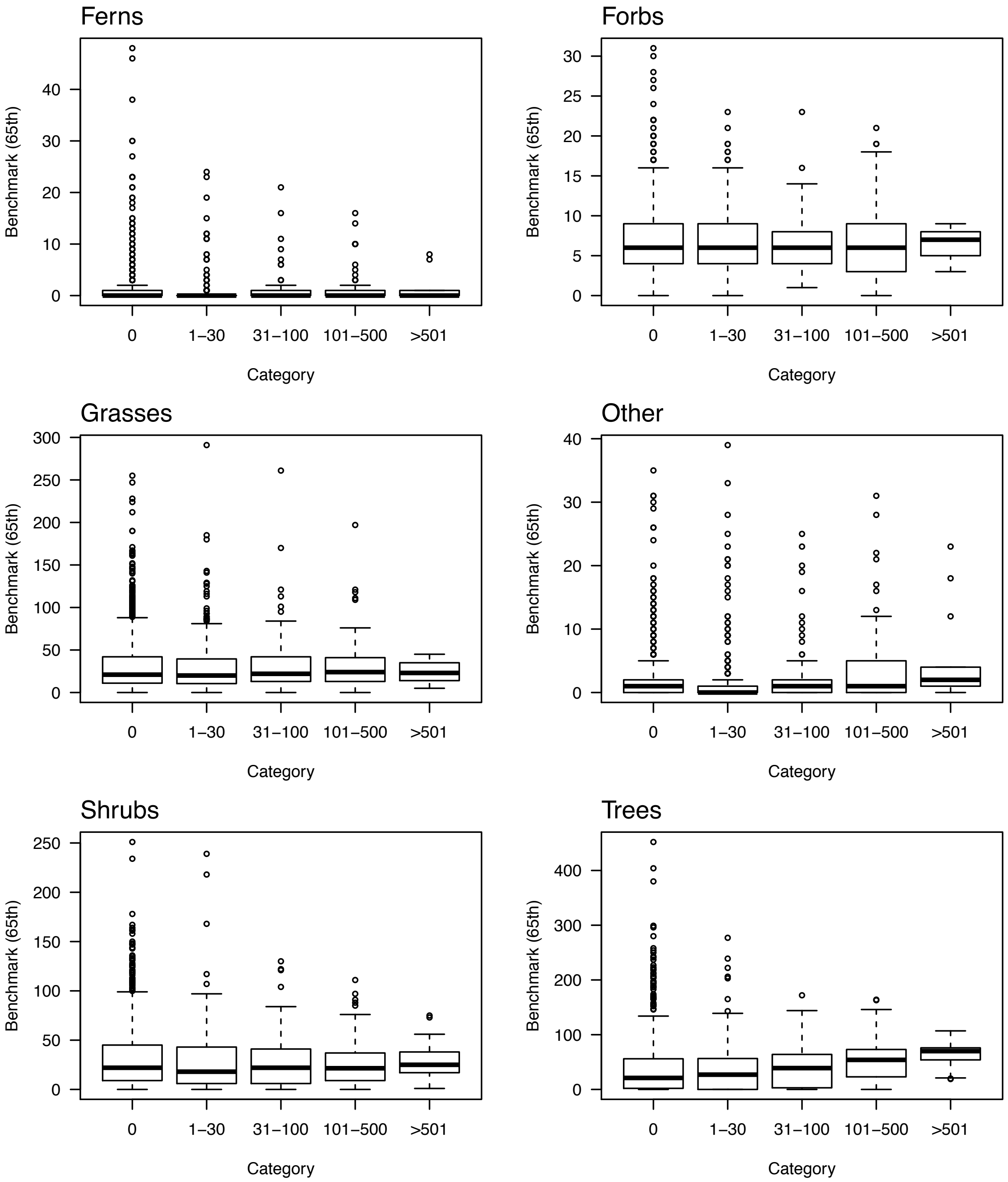

Fig. S5. Estimated native species cover benchmarks (65th percentiles) plotted against number of plots in each combination of bioregion and vegetation class.

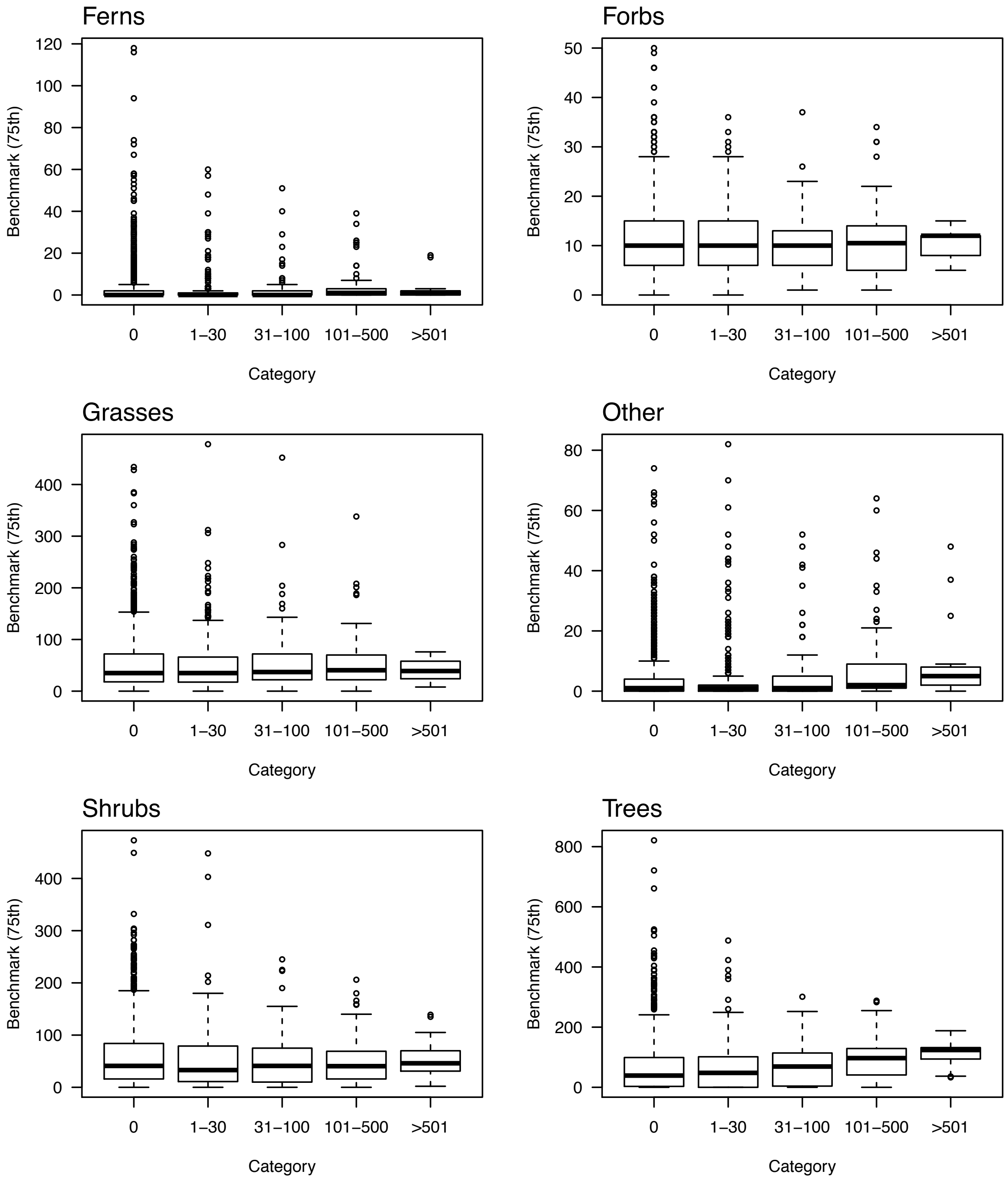

Fig. S6. Estimated native species cover benchmarks (75th percentiles) plotted against number of plots in each combination of bioregion and vegetation class.
